# Birthweight and Childhood Cancer: Preliminary Findings from the International Childhood Cancer Cohort Consortium (I4C)

**DOI:** 10.1111/ppe.12193

**Published:** 2015-05-19

**Authors:** Ora Paltiel, Gabriella Tikellis, Martha Linet, Jean Golding, Stanley Lemeshow, Gary Phillips, Karen Lamb, Camilla Stoltenberg, Siri E Håberg, Marin Strøm, Charlotta Granstrøm, Kate Northstone, Mark Klebanoff, Anne-Louise Ponsonby, Elizabeth Milne, Marie Pedersen, Manolis Kogevinas, Eunhee Ha, Terence Dwyer

**Affiliations:** 1 Department of Hematology, Braun School of Public Health, Hadassah-Hebrew UniversityJerusalem, Israel; 2 Department of Environmental and Genetic Epidemiology, Murdoch Children’s Research Institute, Royal Childrens Hospital, University of MelbourneMelbourne, Australia; 3 Centre for Physical Activity and Nutrition, Deakin UniversityBurwood, Australia; 4 Menzies Research Institute, University of TasmaniaHobart, Tasmania, Australia; 5 Telethon Kids Institute, University of Western AustraliaPerth, Western Australia, Australia; 6 Radiation Epidemiology Branch, Division of Cancer Epidemiology and Genetics, National Cancer Institute, National Institutes of HealthBethesda, MD, UK; 7 Division of Biostatistics, College of Public Health, The Ohio State UniversityColumbus, Ohio, UK; 8 Division of Biostatistics, The Ohio State University Center for BiostatisticsColumbus, Ohio, UK; 9 The Research Institute at Nationwide Children’s Hospital, The Ohio State University College of MedicineColumbus, OH, UK; 10 Centre for Child & Adolescent Health, School of Social & Community Medicine, University of BristolBristol, UK; 11 ALSPAC (Children of the 90s), School of Social and Community Medicine, University of BristolBristol, UK; 12 Norwegian Institute of Public HealthOslo, Norway; 13 Department of Global Public Health and Community Care, University of BergenBergen, Norway; 14 Department of Epidemiology Research, Center for Fetal Programming, Statenserum InstituteCopenhagen, Denmark; 15 Centre for Research in Environmental Epidemiology (CREAL)Barcelona, Spain; 16 Universitat Pompeu FabraBarcelona, Spain; 17 IMIM (Hospital del Mar Research Institute)Barcelona, Spain; 18 CIBER Epidemiología y Salud Pública (CIBERESP)Madrid, Spain; 19 U823, Team of Environmental Epidemiology Applied to Reproduction and Respiratory Health, Institute Albert Bonniot, INSERM (National Institute of Health and Medical Research)Grenoble, France; 20 International Agency for Research on CancerLyon, France; 21 Department of Nutrition, National School of Public HealthAthens, Greece; 22 School of Medicine, Ewha Medical Research Center, Department of Preventive Medicine, Ewha Womans UniversitySeoul, Korea

**Keywords:** Childhood cancer, leukemia, cohort studies, pooled analysis.

## Abstract

**Background:**

Evidence relating childhood cancer to high birthweight is derived primarily from registry and case–control studies. We aimed to investigate this association, exploring the potential modifying roles of age at diagnosis and maternal anthropometrics, using prospectively collected data from the International Childhood Cancer Cohort Consortium.

**Methods:**

We pooled data on infant and parental characteristics and cancer incidence from six geographically and temporally diverse member cohorts [the Avon Longitudinal Study of Parents and Children (UK), the Collaborative Perinatal Project (USA), the Danish National Birth Cohort (Denmark), the Jerusalem Perinatal Study (Israel), the Norwegian Mother and Child Cohort Study (Norway), and the Tasmanian Infant Health Survey (Australia)]. Birthweight metrics included a continuous measure, deciles, and categories (≥4.0 vs. <4.0 kilogram). Childhood cancer (377 cases diagnosed prior to age 15 years) risk was analysed by type (all sites, leukaemia, acute lymphoblastic leukaemia, and non-leukaemia) and age at diagnosis. We estimated hazard ratios (HR) and 95% confidence intervals (CI) from Cox proportional hazards models stratified by cohort.

**Results:**

A linear relationship was noted for each kilogram increment in birthweight adjusted for gender and gestational age for all cancers [HR = 1.26; 95% CI 1.02, 1.54]. Similar trends were observed for leukaemia. There were no significant interactions with maternal pre-pregnancy overweight or pregnancy weight gain. Birthweight ≥4.0 kg was associated with non-leukaemia cancer among children diagnosed at age ≥3 years [HR = 1.62; 95% CI 1.06, 2.46], but not at younger ages [HR = 0.7; 95% CI 0.45, 1.24, *P* for difference = 0.02].

**Conclusion:**

Childhood cancer incidence rises with increasing birthweight. In older children, cancers other than leukaemia are particularly related to high birthweight. Maternal adiposity, currently widespread, was not demonstrated to substantially modify these associations. Common factors underlying foetal growth and carcinogenesis need to be further explored.

Over 50 years ago, MacMahon and Newill^1^ suggested that birthweight may be linked to childhood cancer risk. This putative association was subsequently examined in diverse geographical settings, mainly in case–control studies. Early studies focused on childhood cancer mortality^1^–^3^ while later investigations, summarised in two meta-analyses,^4^,^5^ primarily addressed the association between birthweight and the incidence of acute leukaemia, or its main subtypes, acute lymphoblastic (ALL), and acute myeloid leukaemia (AML). Evidence from these studies supports an overall weakly to moderately increased risk of ALL among children with high birthweight (generally defined as ≥4.0 kg), or a linear association with each kilogram birthweight increment,^4^,^5^ although some studies have had null or negative findings.,^6^–^8^ and the influence of birthweight on AML is less consistent.

Evidence regarding non-leukaemia cancers points to higher risks of renal (notably Wilms), embryonal and specific Central Nervous System (CNS) tumours^9^–^11^ with high birthweight. For some cancers, non-linear models best describe the association with birthweight and, for hepatic tumours (notably hepatoblastoma), a negative association has been observed.^11^

More recent research has emphasised the role of accelerated foetal growth (taking into account factors such as gestational age (GA)), rather than birthweight *per se*, as a determinant of childhood cancer.^12^–^16^ Among these studies are recent pooled analyses of case–control studies.^11^,^17^,^18^ Adjustment for GA may change both the magnitude and the precision of relative risk estimates.^11^

Fetal growth is determined by both environmental and genetic factors^19^ and is influenced by maternal attributes, notably height, parity, diabetes and other metabolic factors, smoking, socioeconomic status, and ethnicity.^20^,^21^ Moreover, maternal pre-pregnancy overweight^22^,^23^ and excess pregnancy weight gain^23^ are increasingly recognised determinants of large-for-GA babies. Current maternal obesity trends portend an increased proportion of these infants,^24^ and a possible concomitant rise in metabolic and cardiovascular morbidity for the offspring.^25^ However, the potential consequences of maternal adiposity for childhood cancer have rarely been considered.^26^ In contrast to a wealth of information regarding determinants of foetal growth, risk factors for childhood cancer are largely unknown. Controversy remains as to whether the association between birthweight and childhood cancer varies, for example, by age at diagnosis.

The International Childhood Cancer Cohort Consortium (I4C)^27^ provides a platform to examine cancer risk factors using pooled data collected prior to disease onset. This, combined with the prospect of evaluating the contribution of a rich set of covariates, affords an opportunity to obtain a deeper and less biased understanding of the association between birthweight and childhood cancer. Our aims were to re-examine this association, taking into account GA and other covariates, and to explore the potential modifying effects of age at diagnosis and maternal anthropometric measures.

## Methods

### The I4C

I4C was established in 2005 to address the lack of prospective, adequately powered studies investigating the aetiology of childhood cancer. The initial collaboration involved 11 international birth/infant cohorts ranging from ∼11 000 to 100 000 participants at various stages of recruitment or follow-up.^27^ Additional cohorts have since joined. This report involves the pooling of data from (alphabetically): the Avon Longitudinal Study of Parents and Children (ALSPAC, UK), the Collaborative Perinatal Project (CPP, USA), the Danish National Birth Cohort (DNBC, Denmark), the Jerusalem Perinatal Study (JPS, Israel), the Norwegian Mother and Child Cohort Study (MoBa, Norway), and the Tasmanian Infant Health Survey (TIHS, Australia). Data from all cohorts were transferred to the I4C International Data Coordinating Center (IDCC) at the Murdoch Childrens Research Institute (Australia). Harmonisation and pooling of data from the six cohorts was undertaken at the I4C IDCC and involved creating variables that would allow for amalgamation of the available data across all six cohorts (Please see supporting information Appendix S1 for description of participating cohorts, references, ethical issues, and harmonisation strategies).

### Study design and population

We performed a pooled cohort study, identifying and including all cancer cases from 380 000 livebirths in the six participating cohorts. The dataset includes all livebirths for ALSPAC, CPP, and TIHS. As per Consortium agreements with the I4C, a random 10% sample of non-cases from MoBa and DNBC rather than the entire cohorts were included. Offspring from the JPS cohort were included if their GA was recorded (from mothers’ pre- or postpartum interviews), comprising all those born 1974–1976, and a subset born 1964–1973 (total *n* = 20 944). The pooled dataset thus comprises 112 781 livebirths, after excluding multiple births (due to their high rate of low birthweight)^28^ and children with Down syndrome (due to their particularly high risk of childhood leukaemia)^29^ (Table 1).

**Table 1:** Descriptive maternal, paternal, and offspring characteristics of the six I4C member cohorts included in the pooled dataset

	ALSPAC	CPP	DNBC	JPS	MoBa	TIHS	Total
**Recruitment years**	1991–1992	1959–1965	1996–2002	1964–1976	1999–2009	1987–1995	1959–2007
**Singleton livebirths with no DS**	13 664	50 342	8603	20 313	10 497	9 362	112 781
**Years of follow-up**	14.9	5.6	11	15	4.4	14.7	9.9
Mean (range)	(0.5–15)	(0.0–8.0)	(8.3–14.0)	(15.0–15.0)	(0.5–10.1)	(12.7–15.0)	(0.0–15.0)
**Maternal age (years)**							
Mean ± SD	28.0 ± 5.0	24.1 ± 5.9	30.5 ± 4.2	27.3 ± 5.4	30.2 ± 4.6	23.6 ± 4.4	26.2 ± 5.9
*Missing (%)*	*0 (0.0)*	*0 (0.0)*	*2 (0.02)*	*77 (3.8)*	*10 (0.1)*	*0 (0.0)*	*89 (0.08)*
**Married or cohabitating, n (%)**	9 588 (70.2)^a^	38 658 (76.8)	8094 (94.1)	20 142 (99.2)	9 591 (91.4)	7 318 (78.2)	93 389 (82.8)
*Missing (%)*	*861 (6.3)*	*2 (0.01)*	*359 (4.2)*	*114 (0.6)*	*591 (5.6)*	*32 (0.3)*	*1 590 (1.7)*
**Mother completed 12 or more years of education, n (%)**	4 286 (31.4)^d^	20 767 (41.3)	4097 (47.6)	9 866 (48.6)	6 246 (59.5)	1 690 (18.0)	46 952 (41.6)
*Missing (%)*	*1 536 (11.2)*	*122 (0.24)*	*2349 (27.3)*	*358 (1.8)*	*595 (5.7)*	*21 (0.2)*	*4 981 (4.4)*
**Maternal prenatal smoking, *n* (%)**	3 530 (25.8)	23 269 (46.2)	2 193 (25.5)	2 568 (12.6)	925 (8.8)	5 023 (53.6)	37 508 (33.3)
*Missing (%)*	*1 639 (12.0)*	*263 (0.5)*	*9 (0.1)*	*193 (1.0)*	*2 295 (21.9)*	*16 (0.2)*	*4 415 (3.9)*
**Passive smoking at home, prenatal, *n* (%)**^b^	5 362 (39.2)	n/a	5584 (64.9)	7 438 (36.6)	770 (7.3)	5 242 (56.0)	24 396 (39.1)
*Missing (%)*	*1 859 (13.6)*		*3005 (34.9)*	*3 828 (18.8)*	*1 397 (13.3)*	*20 (0.2)*	*10 109 (16.2)*
**Parity, *n* (%)**							
No prior livebirth	1 377 (10.1)	1 142 (2.3)	n/a	n/a	1 239 (11.8)	n/a	3 758 (3.6)
No prior pregnancy	4 263 (31.2)	14 187 (28.2)	3861 (44.9)	6 249 (30.8)	3 229 (30.8)		31 789 (30.7)
1–2	6 090 (44.6)	19 768 (39.3)	4117 (47.9)	8 713 (42.9)	4 960 (47.2)		43 648 (42.2)
≥3	733 (5.4)	15 191 (30.2)	261 (3.0)	5 279 (26.0)	292 (2.8)		21 753 (21.0)
*Missing (%)*	*1 201 (8.8)*	*54 (0.1)*	*364 (4.2)*	*76 (0.4)*	*777 (7.4)*		2 472 (2.4)
**Maternal pre-pregnancy BMI, kg/m^2^**							
Mean ± SD	22.9 ± 3.8	22.7 ± 4.3	23.6 ± 4.4	22.1 ± 3.1	24.0 ± 4.2	23.2 ± 4.8	22.9 ± 4.1
*Missing (%)*	*2 396 (17.5)*	*4 372 (8.7)*	*493 (5.7)*	*6 010 (29.6)*	*835 (7.9)*	*3 113 (33.2)*	*17 219 (15.3)*
**Maternal pregnancy weight gain, kg**							
Mean ± SD	12.5 ± 4.7	9.8 ± 4.8	15.1 ± 5.8	11.2 ± 4.3	14.9 ± 5.8	13.9 ± 6.4	11.5 ± 5.4
*Missing (%)*	*1 573 (11.5)*	*3 816 (7.6)*	*1947 (22.6)*	*5 679 (27.6)*	*2 550 (24.3)*	*1 704 (18.2)*	*17 269 (15.3)*
**Maternal height, cm**							
Mean ± SD	163.9 ± 6.7	160.9 ± 6.9	168.8 ± 6.0	162.0 ± 6.0	168.0 ± 5.9	162.2 ± 7.3	162.9 ± 7.2
*Missing (%)*	*1 717 (12.6)*	*3 740 (7.4)*	*361 (4.2)*	*4 678 (23.0)*	*660 (6.3)*	*2 452 (26.2)*	*13 608 (12.1)*
**Maternal DM, *n* (%)**						n/a	
**Pre-existing**	442 (3.2)^c^	264 (0.5)	25 (0.3)	36 (0.2)	55 (0.5)		882 (0.8)
**Gestational**	55 (0.4)	127 (0.2)	139 (1.6)	110 (0.5)	85 (0.8)		516 (0.5)
*Missing (%)*	*1 585 (11.6)*	*129 (0.3)*	*0 (0.0)*	*90 (0.4)*	*0 (0.0)*		*1 804 (1.7)*
**Any previous miscarriage**, ***n* (%)**	2 719 (19.9)	9 031 (17.9)	1563 (18.2)	4 416 (21.7)	1 925 (18.3)	n/a	19 654 (19.0)
*Missing (%)*	*961 (7.0)*	*0 (0)*	*365 (4.2)*	*104 (0.5)*	*777 (7.4)*		2 207 (2.1)
**Paternal age (years)**							
Mean ± SD	30.7 ± 5.8	28.2 ± 7.0	32.8 ± 5.1	30.9 ± 6.5	32.7 ± 5.3	26.5 ± 5.6	29.8 ± 6.6
*Missing (%)*	*2 269 (16.6)*	*12 795 (25.4)*	*123 (1.4)*	*654 (3.2)*	*47 (0.4)*	*215 (2.3)*	*16 103 (14.3)*
**Father completed at least 12 years of education, *n* (%)**	5 151 (37.7)^d^	19 209 (38.2)	2700 (31.4)	10 828 (53.3)	4 782 (45.6)	1 624 (17.3)	44 294 (39.3)
*Missing (%)*	*2 012 (14.7)*	*9 651 (19.2)*	*2541 (29.5)*	*418 (2.1)*	*883 (8.4)*	*865 (9.2)*	*16 370 (14.5)*
**Gestational age, weeks**							
Mean ± SD	39.5 ± 1.9	39.4 ± 3.1	40.1 ± 1.7	39.7 ± 2.2	39.5 ± 1.8	38.8 ± 2.6	39.5 ± 2.6
*Missing (%)*	*0 (0.0)*	*329 (0.6)*	*0 (0.0)*	*0 (0.0)*	*47 (0.4)*	*29 (0.3)*	*405 (0.4)*
**Gender, male *n* (%)**	7 052 (51.6)	25 461 (50.6)	4367 (50.8)	10 485 (51.6)	5 274 (50.2)	6 673 (71.3)	59 312 (52.6)
*Missing (%)*	*2 (0.01)*	*81 (0.2)*	*0 (0.0)*	*0 (0.0)*	*0 (0.0)*	*0 (0.0)*	*93 (0.1)*
**Birthweight, grams**							
Mean ± SD	3 410 ± 551	3 177 ± 531	3599 ± 549	3 260 ± 508	3 604 ± 562	3 195 ± 751	3 293 ± 577
*Missing (%)*	*172 (1.3)*	*192 (0.4)*	*32 (0.4)*	*52 (0.3)*	*20 (0.2)*	*0 (0.0)*	*468 (0.4)*
**Placental weight, grams**							
Mean ± SD	652 ± 138	437 ± 94	663 ± 148	n/a	676 ± 149	613 ± 161	531 ± 163
*Missing (%)*	*8 103 (59.3)*	*7 864 (15.6)*	*288 (3.3)*		*325 (3.1)*	*149 (1.6)*	*16 729 (18.1)*
**First born, *n* (%)**	5 500 (40.2)	14 187 (28.2)	3861 (44.9)	6 248 (30.8)	4 468 (42.6)	44 387 (46.9)	38 651 (34.3)
*Missing (%)*	*1 090 (8.0)*	*54 (0.1)*	*364 (4.2)*	*76 (0.4)*	*777 (7.4)*	*11 (0.1)*	*2 372 (2.1)*
**Length at birth, cm**							
Mean ± SD	50.7 ± 2.4	49.9 ± 2.7	52.3 ± 2.5	n/a	50.4 ± 2.4	48.8 ± 3.4	50.2 ± 2.9
*Missing (%)*	*3 399 (24.9)*	*1 515 (3.0)*	*81 (0.9)*		*387 (3.7)*	*221 (2.4)*	*5 603 (6.1)*

ALSPAC = the Avon Longitudinal Study of Parents and Children (UK); CPP = the Collaborative Perinatal Project (USA); DNBC = the Danish National Birth Cohort (Denmark); DS = Down syndrome; JPS = the Jerusalem Perinatal Study (Israel); MoBa = the Norwegian Mother and Child Cohort Study (Norway); n/a = data not collected/provided; TIHS = the Tasmanian Infant Health Survey (Australia).

a Concerned marriage only.

b Passive smoking defined as any exposure to smoke at home by partner or others living in the home.

c Includes glycosuria.

d Educational qualifications obtained were used as a proxy – but by law the school leaving age was 16 at the earliest.

Note that if a subject characteristic was n/a for a particular cohort, then the percentage in the ‘Total’ column is based on total number of observations without including that cohort in the summary statistic.

### Cancer ascertainment

Childhood cancer (diagnosed <15 years of age) was ascertained by linkage to national registries for ALSPAC, DNBC, JPS, and MoBa. For TIHS, linkage was with the Tasmanian Cancer Registry. Cancer cases for CPP were identified via examination of diagnostic summaries and other indirect methods such as identifying children reported in previous investigations of cancer and x-ray exposure, and manually reviewing death records for children with birthweight ≥1500 g who survived the first week of life. Each potential cancer diagnosis was reviewed by two board-certified paediatricians.

Tumours were classified into four main groups based on the International Classification of Diseases (ICD)-0 Third Edition:^30^ all cancers (C-code 42), leukaemia (morphology codes 9800–9941), ALL (codes 9820–9827, 9850), and non-leukaemia cancer (C-code 42, excluding 9800–9991). Small numbers of AML and specific solid tumours across the six cohorts precluded analysis of individual cancer subtypes besides ALL.

### Birthweight metrics

Birthweight was analysed using three approaches: first, dichotomised as ≥4.0 kg vs. <4.0 kg. The second approach took into account differing birthweight distributions across populations and time. For example the 90th percentile of birthweight was as follows: ALSPAC: 4129, CPP: 3827, DNBC: 4320, JPS: 3880, MoBa: 4260, and TIHS: 4030 g. To explore whether the heaviest newborns in each cohort, regardless of absolute weight, were at higher risk of cancer, we chose membership in the top decile as the ‘exposed’ group while the lower 90% of children comprised the reference group. Finally, birthweight was assessed as a continuous variable in 0.5 and 1.0 kg increments.

### Covariates and potential confounders

A number of variables previously shown to be associated with birthweight or childhood cancer were assessed as potential confounders or effect modifiers. These included:

1. Maternal factors: age at time of index child’s birth (years); married/cohabitating at time of enrolment (yes/no); at least 12 years of education completed (yes/no); any smoking during pregnancy (yes/no); exposure to any smoking at home during pregnancy (yes/no); parity – defined as the number of previous livebirths (for all cohorts except ALSPAC and DNBC that includes number of previous pregnancies and stillbirths), grouped as 0/1–2/≥ 3; pre-existing or gestational diabetes (yes/no); pre-pregnancy body mass index [BMI = weight (kg)/height (m^2^)]; and total pregnancy weight gain (kg).
2. Factors relating to the index child: GA (weeks), determined by date of last menstrual period (or ultrasound in a subgroup from MoBa and ALSPAC); sex; first born (yes/no), birth length (cm); placental weight (g); and age at diagnosis of primary cancer (years).
3. Paternal factors: age at time of index child’s birth (years), and completion of at least 12 years of education (yes/no).

### Follow-up time

Children in the ALSPAC and JPS cohorts were followed to at least 15 years of age, or censored at date of death. Follow-up of children in DNBC and MoBa is ongoing. Children within these cohorts without cancer are assumed to have been followed to the point of last linkage to their national registries: 1 September 2011 and 31 December 2009, respectively. For TIHS, in the absence of systematic follow-up of cohort members, non-cases were deemed to be followed to the last date of diagnosis of a case in the Tasmanian Cancer Registry (28 September 2008), when the youngest child was aged 12.73 years. Follow-up time for the CPP was calculated as the number of months from date of birth (or age 1 week) to the last recorded visit, for a maximum of 8 years.

### Missing data

Missing covariate data among the cohorts ranged from 0% to nearly 40% (see Table 1). In order to construct multivariable models with maximal sets of covariates, we used chained multiple imputation to impute 20 complete datasets.^31^ Cox regression was performed separately on each imputed dataset and the results pooled into a single multiple imputation result. We used truncated linear regression to impute missing continuous variables (paternal age, maternal height, pregnancy weight gain, and pre-pregnancy BMI) where the imputations are limited to lower and upper boundaries set at the minimum and maximum values of non-missing observations. Logistic regression was used to impute missing dichotomous variables (first born and maternal smoking). Variables used to impute missing data were maternal age, GA, birthweight, sex of child, and cohort.

### Statistical analysis

We report hazard ratios (HR) and 95% confidence intervals (CI) from Cox proportional hazards regression models. All models were stratified by cohort. Model 1 was unadjusted (birthweight was the only independent variable). Model 2 adjusted for GA and child’s sex. Model 3 was a parsimonious multivariable model adjusted for GA, child’s sex, as well as different combinations of covariates for each cancer outcome, chosen as follows:

Starting with all confounders in the model, we removed variables one at a time (beginning with the variable with the largest P-value, so long as that variable no longer changed the coefficients for birthweight or the other covariates in the model by >15% in either direction) using the multiple imputation dataset. Once removed, a variable could not re-enter the model.

Schoenfeld residuals were used to assess the proportional hazard assumption with all covariates entered into the model, first, on the original data containing missing observations and, second, after imputing the missing data. Proportionality assumptions were met in both.^32^ We assessed the linearity of continuous variables in the log-hazard using the method of fractional polynomials.^33^ Paternal age, determined to be non-linear, was transformed to a quadratic expression.

To assess the possibility of effect modification by maternal anthropometric measurements, we introduced interaction terms of birthweight × maternal pre-pregnancy BMI using a cut-off of normal or underweight (<25 kg/m^2^) vs. overweight (≥25 kg/m^2^).^34^ In separate models, we introduced an interaction term of birthweight × pregnancy weight gain, dichotomised according to the Institute of Medicine recommendation (based on a healthy BMI) of ≤16 kg vs. >16 kg.^35^

To determine whether the birthweight–cancer relation varied by age at diagnosis, we used a time-varying coefficient approach, allowing for the estimation of two HRs, one before a particular age at diagnosis and one after. This time indicator variable is zero before the relevant age and one afterwards. To test the sensitivity of these results to changes in the indicator time variable, we ran the analyses for 3, 4, 5, 6, and 7 years. The results indicated that the HRs for birthweight were significantly different before and after age 3 years, then fairly stable for years 4 to 7 (data not shown). We thus retained the cut-off at age 3 for our analysis.

To assess heterogeneity effects by cohort, we generated random-effects (shared frailty) Cox models. The results were similar to those obtained using a stratified analysis with each cohort serving as a stratum that we report herein.

Analyses were conducted using Stata Statistical Software, Version 12.1 (StataCorp, College Station, TX, USA).

## Results

Table 1 presents the cohort-specific characteristics of mothers, fathers, and index children. Mean maternal age ranged from 23.6 [standard deviation (SD) 4.4] years (TIHS) to 30.5 (SD 4.3) years (DNBC), with paternal age showing similar variation. Scandinavian mothers were the tallest, on average, yet maternal BMI was fairly consistent across studies. Mean pregnancy weight gain varied from 9.8 kg (4.80) (CPP) to 14.9 (5.8) kg (MoBa). Active maternal smoking during pregnancy ranged from <10% (MoBa) to just over 50% (TIHS). Mean birthweights were higher in the Scandinavian cohorts and lower in CPP.

Table S1 shows the distribution of cancer cases by age and sex and the absolute risks of cancer in each cohort. In total, the pooled analysis included 377 children with cancer, of whom 115 were diagnosed with leukaemia, 98 with ALL, and 262 with non-leukaemia-type cancers, with 54% of cancers occurring among males. Ranges and mean ages at diagnosis varied according to the length of cohort follow-up.

**Figure 1:**
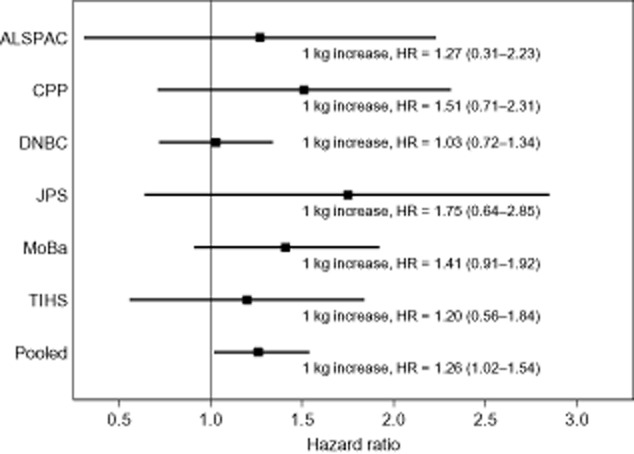
Hazard ratios for any cancer in each cohort and pooled overall for birthweight continuous (per kilogram increase) in a Cox proportional hazards model adjusted for gestational age and sex of child (model 2).

For each cohort, the HRs for all cancers, considering birthweight as a continuous variable (per kilogram) after controlling for GA and child’s sex consistently exceeded 1.0 (Figure 1). Table 2 presents the pooled analysis for birthweight and childhood cancer, leukaemia, ALL, and non-leukaemia cancers. When birthweight was considered as a continuous variable, a significant increased risk of 26% for every kilogram increment in birthweight was observed for all cancers, after adjustment for GA and sex [HR 1.26 (95% CI 1.02, 1.54), *P* = 0.031]. Further adjustment for other covariates (model 3) resulted in similar effect sizes. A 42% increase in risk was also observed for leukaemia, adjusting for GA and child’s sex, with borderline statistical significance. HRs were elevated for children born with birthweight ≥4.0 kg, compared with those with lower birthweight for all cancer outcomes, although the findings were not statistically significant. The pattern was similar when comparing the highest birthweight decile to the lower 90%, per cohort.

**Table 2:** The association between birthweight and childhood cancers, leukaemia, ALL, and non-leukaemia cancers in the pooled dataset

Birthweight metric (*n* cases)	Model 1^b^		Model 2^c^		Model 3^d^	
	HR^a^	95% CI	HR^a^	95% CI	HR^a^	95% CI
Birthweight ≥4.0 kg^e^						
Cancer (377)	1.14	0.88, 1.48	1.19	0.91, 1.55	1.17	0.89, 1.54
Leukaemia (115)	1.25	0.80, 1.96	1.31	0.83, 2.08	1.21	0.74, 1.96
ALL (98)	1.21	0.74, 1.96	1.25	0.76, 2.06	1.21	0.72, 2.04
Non-leukaemia (262)	1.09	0.79, 1.50	1.14	0.82, 1.58	1.11	0.79, 1.56
Top 10% of birthweights in each cohort^f^						
Cancer (377)	1.17	0.85, 1.61	1.22	0.88, 1.69	1.18	0.84, 1.65
Leukaemia (115)	1.25	0.72, 2.19	1.31	0.74, 2.31	1.16	0.63, 2.12
ALL (98)	1.14	0.61, 2.13	1.17	0.62, 2.23	1.08	0.55, 2.14
Non-leukaemia (262)	1.14	0.77, 1.68	1.18	0.80, 1.75	1.14	0.75, 1.71
Continuous birthweight, kg^g^						
Cancer (377)	1.10	0.91, 1.31	**1.26**	**1.02, 1.54**	**1.26**	**1.02, 1.56**
Leukaemia (115)	1.25	0.89, 1.75	1.42	0.98, 2.06	1.35	0.90, 2.02
ALL (98)	1.16	0.81, 1.67	1.29	0.85, 1.93	1.29	0.83, 1.99
Non-leukaemia (262)	1.04	0.83, 1.28	1.19	0.93, 1.52	1.18	0.91, 1.54

a Hazard ratios (95% CI) from a stratified Cox proportional hazard regression using all observations in the pooled dataset. In models 2 and 3 missing observations are imputed using a chained multiple imputation method.

b Model 1 is an unadjusted Cox proportional hazard regression model stratified by cohort in which birthweight is the only independent variable.

c Model 2 is an adjusted Cox proportional hazard regression model stratified by cohort in which birthweight, gestational age, and sex of the child are the independent variables.

d Model 3 is an adjusted Cox proportional hazard regression stratified by cohort in which, for:
Cancer: birthweight hazard ratio is adjusted for gestational age, child’s sex, maternal age, paternal age (rescaled as quadratic), first born, and maternal pre-pregnancy BMI.

Leukaemia: birthweight hazard ratio is adjusted for gestational age, child’s sex, maternal age, total pregnancy weight gain, maternal pre-pregnancy BMI, first born, and any maternal smoking during pregnancy.

ALL: birthweight hazard ratio is adjusted for gestational age, child’s sex, paternal age (rescaled as quadratic), total pregnancy weight gain, and any maternal smoking during pregnancy.

Non-leukaemia cancers: birthweight hazard ratio is adjusted for gestational age, child’s sex, paternal age (rescaled as quadratic), total pregnancy weight gain, first born, and maternal pre-pregnancy BMI.

e Reference group for birthweight ≥4.0 kg is birthweight <4.0 kg.

f The reference group for the top 10% of each cohort is the bottom 90% of each cohort.

g For continuous birthweight, the hazard ratio represents a 1 kg increase in birthweight.

**Figure 2:**
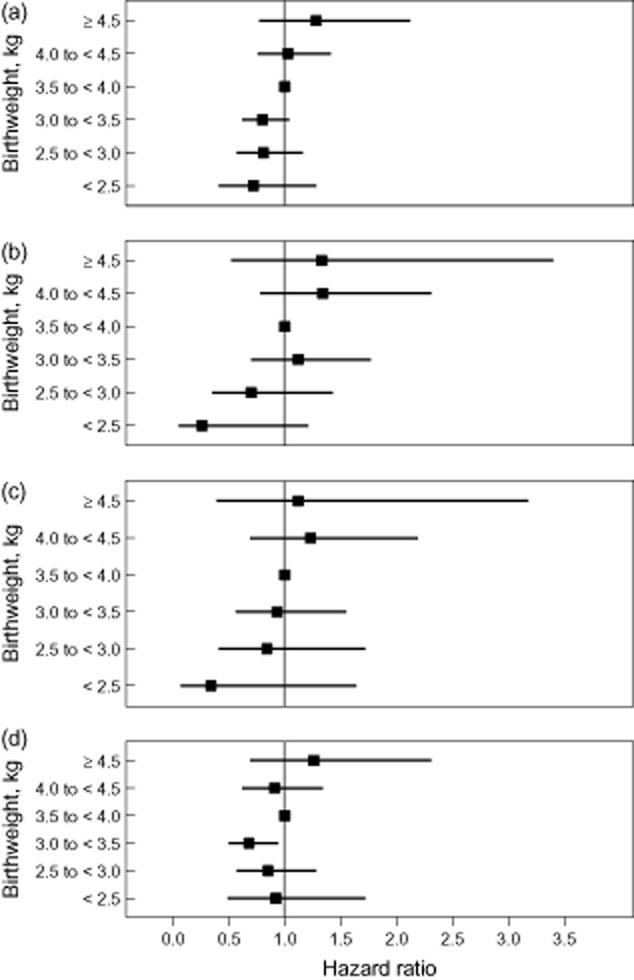
Hazard ratios in the pooled dataset for birthweight* in 500 g increments in Cox proportional hazards models adjusted for gestational age and sex of child (model 2) by cancer type. (a) Cancer; (b) leukaemia; (c) acute lymphoblastic leukemia; (d) non-leukaemia.Spearman rank correlation for all cancers (rho) = 0.943, *P* = 0.005; for all leukaemia rho = 0.886, *P* = 0.019; for ALL rho = 0.943, *P* = 0.005, and for non-leukaemia cancers rho = 0.486, *P* = 0.329.*Birthweight 3.5–<4 kg is the reference category.

Figure 2 shows a monotonic increased risk of all cancers with increasing birthweight [Spearman rank correlation (rho) = 0.943, *P* = 0.005], as well as for leukaemia and ALL, but not for non-leukaemia cancers, in the pooled analysis.

The association between birthweight and childhood cancer differed according to age at diagnosis (Table 3). In models adjusted for GA and sex, a significant association between birthweight, using all metrics, was observed for cancers occurring at or after age 3 years, while HRs were reduced and not statistically significant for younger children. This finding appeared to be driven by non-leukaemia cancers. Although HRs were higher for children diagnosed with leukaemia at or after age 3 years, there was no statistical evidence of time dependency.

**Table 3:** Cancer, leukaemia, ALL, and non-leukaemia cancer hazard ratios in the pooled dataset using a time-varying coefficient for birthweight across two time periods (age at diagnosis <3 vs. ≥3 years old) adjusting for sex and gestational age

		Diagnosed <3 years old			Diagnosed ≥3 years old			Comparison of HRs between time periods, *P*-value
Diagnosis	Birthweight	Cases	HR	95% CI	Cases	HR	95% CI	
Cancer	≥4.0 kg	182	0.84	0.56, 1.27	195	1.60	1.13, 2.26	0.018
	Top 10%		0.80	0.46, 1.39		1.64	1.10, 2.44	0.037
	Continuous		1.08	0.82, 1.42		1.44	1.11, 1.88	0.099
Leukaemia	≥4.0 kg	59	1.08	0.55, 2.13	56	1.56	0.84, 2.88	0.43
	Top 10%		1.08	0.46, 2.54		1.55	0.72, 3.30	0.54
	Continuous		1.29	0.79, 2.11		1.57	0.96, 2.57	0.56
ALL	≥4.0 kg	49	1.02	0.48, 2.15	49	1.49	0.77, 2.88	0.45
	Top 10%		1.07	0.42, 2.73		1.28	0.54, 3.03	0.79
	Continuous		1.23	0.72, 2.11		1.34	0.78, 2.30	0.81
Non-leukaemia	≥4.0 kg	123	0.75	0.45, 1.24	139	1.62	1.06, 2.46	0.020
	Top 10%		0.67	0.33, 1.38		1.68	1.05, 2.68	0.035
	Continuous		0.99	0.71, 1.38		1.39	1.02, 1.91	0.10

Model 2: Stratified Cox proportional hazard regression with a time-varying coefficient for birthweight based on an indicator function for time defined at the age of diagnosis cut-point adjusted for gestational age and sex of the child.

Maternal pre-pregnancy BMI (unadjusted HR 1.01, 95% CI 0.99, 1.04) and pregnancy weight change (unadjusted HR 1.0, 95% CI 0.99, 1.02) were not in themselves associated with childhood cancer risks. We explored potential effect modification by these anthropometric measures on the association between birthweight and the various cancer outcomes, and found no significant interactions. Specifically, HRs, in general, did not substantially differ when we examined the association between birthweight and cancer, leukaemia or non-leukaemic tumours in two strata of maternal pre-pregnancy BMI (top half of Table S2), or in the high and low strata of gestational weight gain. It should be noted that case numbers in each stratum were relatively limited.

## Comment

In this pooled analysis, we provide evidence from prospectively collected data that birthweight, adjusted for GA and sex, is positively associated with increased cancer and leukaemia risks in children. In addition, higher birthweight is particularly associated with non-leukaemia cancer diagnosed at or after age 3 years. Although maternal obesity is associated with high birthweight,^22^,^23^ heightened cancer risks in high birthweight offspring of overweight women or those with excessive pregnancy weight gain were not observed in our exploratory analyses.

For every kilogram increment in birthweight, the HR for cancer was 1.26, similar to that reported in large registry-based studies. The Norwegian Medical Birth and Cancer Registries reported a HR of 1.23 (1.14–1.32)/kg birthweight increase adjusting for GA,^14^ with no modification by age at diagnosis. Recently, a case–control study^9^ (17 698 cases, 172 422 controls) based on registries in four Nordic countries reported odds ratios (OR) of 1.2 and 1.4 for birthweight 4000–4500 and 4500–6000 g, respectively, for all cancers; OR estimates for ALL were also similar to our pooled analysis, with little variation among age groups.^9^ Comparable findings were reported in a large cohort of ethnic Chinese in Singapore.^36^ Among nearly 2 000 000 children identified through the Danish Birth Registry, Westergaard showed a Relative Risk (RR) for ALL of 1.46/kg birthweight increase.^37^ In contrast, a recent large study reporting on a total of 40 326 cases and 86 922 controls from the UK and US showed more modestly elevated ORs of 1.06 per 500 g increment for all cancer. For ALL, reported ORs were 1.08 (UK) and 1.11 (US), the latter adjusted for GA.^11^

In our dataset, high birthweight was strongly associated with non-leukaemia cancers diagnosed at or after the age of 3 years. However, leukaemia risks were not modified by age at diagnosis, echoing findings from a large meta-analysis.^5^ The time-varying pattern of birthweight effects may be due to the fact that many cancer subtypes vary by age of onset and their relation with birthweight may vary. As the I4C cohorts mature, more detailed analyses on specific solid tumours and lymphomas, including those which are usually diagnosed in older children will be possible.

Few investigators have explored the effects of maternal anthropometrics on the birthweight–leukaemia or birthweight–cancer association. McLaughlin and colleagues,^26^ in a case–cohort study, noted an association between birthweight and leukaemia only among infants whose mothers weighed <80 kg. That study lacked data on maternal height, thus overweight *per se*, as measured by BMI, was not addressed. They observed an effect of pregnancy weight gain on ALL risk (RR 1.31), using a cut-off of 14.1 kg; however, no interaction with birthweight was noted. Most women in the I4C cohorts were non-obese, with pregnancy weight gain within the recommended range. However, given worldwide trends in maternal adiposity,^38^ this relation deserves further scrutiny, particularly as our analysis was limited by small numbers.

Moving beyond the established association between accelerated foetal growth and childhood cancer to explanatory mechanisms presents a considerable challenge. The complex contributions of both genetics and the intrauterine environment are illustrated by early observations, even among twins, that the heavier sibling was more likely to develop leukaemia.^3^ Proposed biological explanations include increased risks of somatic mutations related to higher stem cell number in large babies, and growth factor effects (e.g. IGF) on both foetal growth and leukaemogenesis. Early clues suggest that haplotypes in *IGF1* and *IGF2* are related to both high birthweight and ALL risk.^39^ Furthermore, overgrowth syndromes related to abnormal methylation patterns of *IGF* genes have been associated with particular cancers.^40^

Our study’s strengths include prospectively collected data from a wide variety of geographic and temporal settings. All birth and maternal characteristics were ascertained at birth or during pregnancy, minimising recall bias. Most of the contributing cohorts were representative of their respective source populations and cancer cases were derived from the same populations as non-cases. This contrasts with case–control studies, in which (because of low response rates and consequent selection bias) controls may differ substantially from the case population, including in their birthweight distribution.^41^

Given that the correlation between birthweight and birthweight-adjusted-for-GA is not high (kappa = 0.45),^16^ accounting for GA, as we did, is important. In our analysis, adjustment for GA generally resulted in improved precision of the HR estimates.

The I4C platform enabled us to simultaneously examine a wide range of potential confounders (e.g. maternal adiposity, parity, diabetes, and maternal active and passive smoking) unavailable in many previous record-linkage studies. However, the maximally adjusted models did not differ substantially from those which adjusted for child’s sex and GA.

Our study’s limitations include the modest number of cases available for analysis – despite the pooling of six cohorts. This restricted our ability to study subtypes such as AML, as well as specific solid tumours, and provided limited power to study interactions. Missing covariate data necessitated imputation. Subjects in some of the cohorts had not yet reached 15 years of age, so the entire childhood cancer experience of the cohort cannot be fully summarised. Furthermore, methods of cancer ascertainment and follow-up were inconsistent among the cohorts, and for one cohort (TIHS), enrolment was selective.

Pooling data from different cohorts may be problematic due to heterogeneity of observed effects. For instance, variation by ethnicity may occur in the association between *IGF* haplotypes, leukaemia, and birthweight.^41^ Recent pooled analyses have shown substantial heterogeneity in the association between birthweight and cancer across countries.^11^,^18^ In an attempt to diminish the effects of differential birthweight distributions across cohorts, we stratified all models by cohort and performed an analysis taking into account the highest birthweight decile within each cohort. The analysis using birthweight (adjusted for GA and child sex) as a continuous variable showed consistent results across cohorts (Figure 1), and can serve as a simple measure facilitating comparison between large registry-based studies, meta-analyses, and pooled analyses of case–control studies.

In conclusion, evidence has now been added from pooled prospectively collected data spanning six countries on four continents over 50 years, strengthening the observation that increasing birthweight is a risk factor for childhood cancer and leukaemia. Notwithstanding the known association of maternal obesity with high birthweight and potential metabolic and cardiovascular morbidity, our preliminary findings do not support a substantial main effect of maternal adiposity on childhood cancer nor an interaction with birthweight. With accumulating person-years of follow-up, the addition of cancer cases from newer cohorts, and the availability of biological samples, I4C’s future pooled projects will enable further exploration of the roles of pre- and postnatal events, genetics and epigenetics, as well as providing power to discern which cancer subtypes are associated with high birthweight in older children. Further investigations should continue to focus on mechanisms and exposures that jointly influence both foetal growth and malignant transformation.

## Supporting Information

**Table S1.** Distribution and absolute risks of all cancers, leukaemia, acute lymphoblastic leukaemia (ALL), and non-leukaemia cancer by cohort, gender, and age of diagnosis, in the pooled dataset based on singleton births and excluding children with Down syndrome. **Table S2.** Assessment of maternal pre-pregnancy body mass index (BMI) and pregnancy weight gain as possible effect modifiers of the association between childhood cancers, leukaemia, acute lymphoblastic leukaemia (ALL), and non-leukaemia cancers and birthweight adjusting for sex and gestational age in the pooled dataset. **Appendix S1.** Participating I4C-member cohorts, data harmonization and ethics.
