# Supplementary material for: Birthweight and Childhood Cancer: Preliminary Findings from the International Childhood Cancer Cohort Consortium (I4C)
Source: Paediatr Perinat Epidemiol. 2015 May 19;29(4):335–45. doi: 10.1111/ppe.12193 (PMC4690513; doi:10.1111/ppe.12193)
Supplement: Supplementary file 1 [file ppe0029-0335-sd1.zip › for_paediatric_and_perinatal_epidemiologysupplementary_tables_for__resubmission_march_2015.doc]

**Supplemental TABLE 1:** Distribution and absolute risks of all cancers, leukemia, acute lymphoblastic leukemia (ALL), and non-leukemia cancer by cohort, gender, and age of diagnosis, in the pooled dataset based on singleton births and excluding children with Down Syndrome

|  | **ALSPAC** | | **CPP** | | **DNBC** | | **JPS** | | **MoBA** | | **TIHS** | | **Total** | |
| --- | --- | --- | --- | --- | --- | --- | --- | --- | --- | --- | --- | --- | --- | --- |
| **ALL CANCERS** | N | Age at diagnosis  Mean ±SD | N | Age at diagnosis  Mean ±SD | N | Age at diagnosis  Mean ±SD | N | Age at diagnosis  Mean ±SD | N | Age at diagnosis  Mean ±SD | N | Age at diagnosis  Mean ±SD | N | Age at diagnosis  Mean ± SD |
| Boys | 12 | 8.5 ± 4.8 | 27 | 2.9 ± 2.0 | 70 | 3.8 ± 2.7 | 22 | 7.3 ± 4.6 | 56 | 1.9 ± 1.7 | 17 | 7.6 ± 4.8 | 204 | 4.1 ± 3.7 |
| Girls | 10 | 8.5 ± 4.5 | 22 | 3.0 ± 2.3 | 73 | 4.3 ± 2.9 | 16 | 6.1 ± 4.7 | 45 | 2.3 ± 1.8 | 7 | 6.2 ± 3.5 | 173 | 4.1 ± 3.1 |
| Overall | 22 | 8.5 ± 4.6 | 49 | 2.9 ± 2.1 | 143 | 4.1 ± 2.8 | 38 | 6.8 ± 4.7 | 101 | 2.1 ± 1.7 | 24 | 7.2 ± 4.5 | 377 | 4.1 ± 3.5 |
| *Absolute risk (cases per 10,000 person-years)* |  | *1.079* |  | *1.517* |  | *1.533* |  | *1.248* |  | *2.121* |  | *1.745* |  | *1.125* |
| **LEUKEMIA** |  |  |  |  |  |  |  |  |  |  |  |  |  |  |
| Boys | 3 | 6.0 ± 7.0 | 7 | 2.7 ± 1.3 | 22 | 3.8 ± 1.6 | 5 | 6.3 ± 4.1 | 18 | 2.2 ± 1.4 | 3 | 7.3 ± 6.3 | 58 | 3.7 ± 2.9 |
| Girls | 0 |  | 9 | 2.7 ± 2.0 | 26 | 4.1 ± 2.5 | 3 | 1.4 ± 0.4 | 18 | 3.2 ± 1.8 | 1 | 5.2 | 57 | 3.5 ± 2.2 |
| Overall | 3 | 6.0 ± 7.0 | 16 | 2.7 ± 1.7 | 48 | 4.0 ± 2.1 | 8 | 4.5 ± 4.0 | 36 | 2.7 ± 1.7 | 4 | 6.7 ± 5.3 | 115 | 3.6 ± 2.6 |
| *Absolute risk (cases per 10,000 person-years)* |  | *0.147* |  | *0.495* |  | *0.518* |  | *0.263* |  | *0.787* |  | *0.291* |  | *0.397* |

Supplemental Table 1 continued

| **ACUTE LYMPHOBLASTIC LEUKEMIA** |  |  |  |  |  |  |  |  |  |  |  |  |  |  |
| --- | --- | --- | --- | --- | --- | --- | --- | --- | --- | --- | --- | --- | --- | --- |
| Boys | 3 | 6.0 ± 7.0 | 6 | 2.8 ± 1.3 | 21 | 3.8 ± 1.7 | 5 | 6.3 ± 4.1 | 14 | 2.3 ± 1.4 | 1 | 2.7 | 50 | 3.6 ± 2.6 |
| Girls | 0 |  | 5 | 3.0 ± 1.2 | 22 | 4.1 ± 2.4 | 3 | 1.4 ± 0.4 | 17 | 3.2 ± 1.9 | 1 | 5.2 | 48 | 3.5 ± 2.1 |
| Overall | 3 | 6.0 ± 7.0 | 11 | 2.9 ± 1.2 | 43 | 4.0 ± 2.0 | 8 | 4.5 ± 4.0 | 31 | 2.8 ± 1.7 | 2 | 4.0 ± 1.8 | 98 | 3.6 ± 2.4 |
| *Absolute risk (cases per 10,000 person-years)* |  | *0.147* |  | *0.340* |  | *0.464* |  | *0.263* |  | *0.678* |  | *0.145* |  | *0.341* |
| **NON-LEUKEMIA CANCERS** |  |  |  |  |  |  |  |  |  |  |  |  |  |  |
| Boys | 9 | 9.3 ± 4.1 | 20 | 3.0 ± 2.2 | 48 | 3.8 ± 3.1 | 17 | 7.6 ± 4.9 | 38 | 1.8 ± 1.8 | 14 | 7.7 ± 4.8 | 146 | 4.3 ± 4.0 |
| Girls | 10 | 8.5 ± 4.5 | 13 | 3.2 ± 2.4 | 37 | 4.4 ± 3.1 | 13 | 7.1 ± 4.6 | 27 | 1.6 ± 1.5 | 6 | 6.4 ± 3.8 | 116 | 4.4 ± 3.7 |
| Overall | 19 | 8.9 ± 4.2 | 33 | 3.1 ± 2.3 | 95 | 4.1 ± 3.1 | 30 | 7.4 ± 4.7 | 65 | 1.7 ± 1.7 | 20 | 7.3 ± 4.4 | 262 | 4.3 ± 3.9 |
| *Absolute risk (cases per 10,000 person-years)* |  | *0.931* |  | *1.021* |  | *1.014* |  | *0.985* |  | *1.334* |  | *1.454* |  | *0.728* |

**Supplemental Table 2:** Assessment of maternal pre-pregnancy BMI and pregnancy weight gain as possible effect modifiers of the association between childhood cancers, leukemia, ALL and non-leukemia cancers and birthweight adjusting for sex and gestational age in the pooled dataset

| **Stratum** |  | **Birthweight** | **Cancer***  **HR^1^ [95% CI]** | **Leukemia***  **HR^1^ [95% CI]** | **ALL***  **HR^1^ [95% CI]** | **Non-leukemia***  **HR^1^ [95% CI]** |
| --- | --- | --- | --- | --- | --- | --- |
| **Pre-pregnancy BMI < 25kg/m^2^**  **N = 86,354** | **Cases** |  | 268 | 83 | 74 | 185 |
|  |  | **≥ 4,000 g** | 1.18 [0.84,1.65] | 1.30 [0.73,2.29] | 1.35 [0.75,2.45] | 1.12 [0.74,1.71] |
|  |  | **Top 10%** | 1.11 [0.71,1.72] | 1.38 [0.68,2.80] | 1.38 [0.65,2.91] | 0.98 [0.56,1.73] |
|  |  | **Continuous** | 1.23 [0.96,1.56] | 1.44 [0.93,2.22] | 1.38 [0.86,2.21] | 1.14 [0.85,1.53] |
| **Pre-pregnancy BMI ≥ 25kg/m^2^**  **N = 26,249** | **Cases** |  | 109 | 32 | 24 | 77 |
|  |  | **≥ 4,000 g** | 1.18 [0.76,1.84] | 1.38 [0.62,3.05] | 1.12 [0.44,2.81] | 1.11 [0.65,1.89] |
|  |  | **Top 10%** | 1.37 [0.82,2.28) | 1.22 [0.46,3.23] | 0.88 [0.26,3.00] | 1.42 [0.78,2.59] |
|  |  | **Continuous** | 1.29 [0.92,1.81] | 1.45 [0.75,2.79] | 1.17 [0.56,2.42] | 1.23 [0.83,1.84] |
| **Pregnancy weight gain ≤16 kg**  **N = 92,811** | **Cases** |  | 256 | 75 | 61 | 182 |
|  |  | **≥ 4,000 g** | 1.24 [0.86,1.77] | 1.40 [0.73,2.68] | 1.13 [0.53,2.41] | 1.16 [0.74,1.83] |
|  |  | **Top 10%** | 1.19 [0.75,1.88] | 1.33 [0.59,2.99] | 0.97 [0.36,2.63] | 1.13 [0.64,2.00] |
|  |  | **Continuous** | 1.16 [0.90,1.49] | 1.39 [0.88,2.22] | 1.15 [0.70,1.92] | 1.08 [0.80,1.45] |
| **Pregnancy weight gain > 16 kg**  **N = 19,666** | **Cases** |  | 121 | 40 | 37 | 80 |
|  |  | **≥ 4,000 g** | 1.03 [0.68,1.56] | 1.06 [0.51,2.19] | 1.20 [0.56,2.55] | 1.02 [0.60,1.73] |
|  |  | **Top 10%** | 1.08 [0.64,1.83] | 1.03 [0.41,2.59] | 1.11 [0.43,2.87] | 1.11 [0.57,2.14] |
|  |  | **Continuous** | 1.37 [0.95,1.96] | 1.35 [0.72,2.53] | 1.40 [0.72,2.71] | 1.37 [0.89,2.13] |

Legend Supplementary table 2:

*Hazard ratios (95%CI) from a stratified Cox proportional hazard regression model stratified by maternal pre-pregnancy BMI using all observations in the pooled dataset where missing observations are imputed using a chained multiple imputation method.

^1^Referent group for birth weight ≥ 4.0 kg is birthweight < 4.0 kg, the referent group for the top 10% of each cohort is the bottom 90% of each cohort, and for continuous birthweight the hazard ratio represents a 1 kg increase in birthweight.
