# Supplementary material for: Birthweight and Childhood Cancer: Preliminary Findings from the International Childhood Cancer Cohort Consortium (I4C)
Source: Paediatr Perinat Epidemiol. 2015 May 19;29(4):335–45. doi: 10.1111/ppe.12193 (PMC4690513; doi:10.1111/ppe.12193)
Supplement: Supplementary file 1 [file ppe0029-0335-sd1.zip › supporting_information-revised3003.doc]

Supporting information:

*Participating I4C-member cohorts*

The ALSPAC study is a birth cohort of 14,541 pregnancies recruited during 1990 to 1992, selected as a representative sample from the geographical region of Avon and resulting in 14,062 live births.^1,2^ The study website contains details of all the data that are available through a fully searchable data dictionary:

<http://www.bris.ac.uk/alspac/researchers/data-access/data-dictionary/>

The CPP was a prospective, longitudinal study of pregnancies from about 48,000 women enrolled at twelve university sites from 1959 through to 1965. At the termination of the study, the sample consisted of approximately 58,000 live born infants.^3-5^ Children were followed to age 7 or 8 years. The DNBC is a population-based cohort of 101,042 pregnancies resulting in 96,840 live births from mothers who were recruited between 1996 and 2002 across Denmark.^6^ The JPS is a population-based cohort that included all births to Israeli residents of West Jerusalem during 1964 to 1976. The study collected data on a total of 92,408 live- and stillbirths and their parents.^7^ The MoBa is a population-based pregnancy cohort consisting of 108 487 live-born infants whose mothers were recruited from across Norway from 1999 to 2009.^8^ The TIHS is a birth cohort constructed between 1988 and 1995 to examine the risk associated with Sudden Infant Death Syndrome (SIDS). The cohort comprises 10,628 live births, including all multiple births, that represent a one in five sample of all births considered at higher risk of SIDS in Tasmania, Australia.^9^

Infants included in the TIHS cohort we selected based on a predictive score for Sudden Infant Death Syndrome (SIDS) which included maternal age, neonatal gender, birthweight and season of birth (March-April, May-June, August-February), duration of second stage of labor and intention to breast feed. As noted, male gender was a selection factor and smoking in these mothers was quite prevalent due to their education levels and young age.

Data harmonization: Maternal education took into account the different education systems across the cohorts. In order to pool data on this variable we converted each level of education into years of schooling and then derived the “mother completed at least 12 years of education” variable based on each individual having completed the equivalent of a secondary school education. Harmonization of maternal prenatal smoking took into account  reports of smoking at various stages in pregnancy. For JPS and CPP there was a variable pertaining to any maternal smoking in pregnancy. For ALSPAC we amalgamated a set of variables regarding reports of pregnancy into one derived variable:: “Maternal prenatal smoking” was defined as ‘Yes’ if smoking in trimester 1 or trimester 2 or trimester 3 was reported as Yes. In DNBC quantity of cigarettes smoked per day was reported; all those who reported smoking were categorizes as smokers, while nonsmokers reported smoking no cigarettes. Maternal smoking is available from interview 1 (around week 12) and from interview 2 (around week 30). For MoBa report of maternal smoking was between 17 and 30 weeks gestation. For THIS smoking was queried regarding three trimesters. We then derived the variable “Prenatal smoking” which was defined as ‘yes’ if number of cigarettes smoked during trimester 1 or 2 or 3 was >0. Similarly, exposure to passive smoking was defined (for cohorts with available data) as father smoking during the pregnancy (JPS), and partner OR other household member for ALSPAC, MoBa and THIS.

*Ethics and approvals for data pooling*

1. For ALSPAC: Ethical approval for the study was obtained from the ALSPAC Ethics and Law Committee and the Local Research Ethics Committees.
2. Data from the CPP are publicly available with identifying information removed.
3. The participants in the DNBC provided written informed consent on behalf of their children. The Regional Scientific Ethics Committee for the municipalities of Copenhagen and Frederiksberg approved all study protocols (case no: (KF) 01-471/94), and all procedures were in accordance with the Declaration of Helsinki.
4. Pooling of JPS data with the I4C, with all identifiers removed, was approved by the institutional review board (Helsinki Committee) of Hadassah University Hospital.
5. Participation in MoBa is based on informed consent. MoBa is approved by the Norwegian Data Inspectorate and the Regional Committee for Medical and Health Research Ethics South East. In addition, the current sub-study in I4C, including linkage with the Cancer Registry of Norway, is approved by the Regional Committee for Medical and Health Research Ethics South East.

6. Parents in the Tasmanian Infant Health Survey (TIHS) provided written consent on behalf of their children. The TIHS and participation of the TIHS in the I4C was approved by the Southern Tasmanian Human Ethics Research Committee.

Supplemental References

1. Boyd A, Golding J, Macleod J, Lawlor DA, Fraser A, Henderson J, et al. Cohort Profile: the 'children of the 90s'--the index offspring of the Avon Longitudinal Study of Parents and Children. International Journal of Epidemiology 2013; 42:111-127.

2. Golding J, Pembrey M, Jones R. ALSPAC--the Avon Longitudinal Study of Parents and Children. I. Study methodology. Paediatric and Perinatal Epidemiology 2001; 15:74-87.

3. Klebanoff MA. The Collaborative Perinatal Project: a 50-year retrospective. Paediatric and Perinatal Epidemiology 2009; 23:2-8.

4. Klebanoff MA, Read JS, Mills JL, Shiono PH. The risk of childhood cancer after neonatal exposure to vitamin K. New England Journal of Medicine 1993; 329:905-908.

5. Shiono PH, Chung CS, Myrianthopoulos NC. Preconception radiation, intrauterine diagnostic radiation, and childhood neoplasia. Journal of the National Cancer Institute 1980; 65:681-686.

6. Olsen J, Melbye M, Olsen SF, Sorensen TI, Aaby P, Andersen AM, et al. The Danish National Birth Cohort-its background, structure and aim. Scandinavian Journal of Public Health 2001; 29:300-307.

7. Harlap S, Davies AM, Deutsch L, Calderon-Margalit R, Manor O, Paltiel O, et al. The Jerusalem Perinatal Study cohort, 1964-2005: methods and a review of the main results. Paediatric and Perinatal Epidemiology 2007; 21:256-273.

8. Magnus P, Irgens LM, Haug K, Nystad W, Skjaerven R, Stoltenberg C. Cohort profile: the Norwegian Mother and Child Cohort Study (MoBa). International Journal of Epidemiology 2006; 35:1146-1150.

9. Dwyer T, Ponsonby AL, Blizzard L, Newman NM, Cochrane JA. The contribution of changes in the prevalence of prone sleeping position to the decline in sudden infant death syndrome in Tasmania. Journal of American Medical Association 1995; 273:783-789.
